# Speech and Language Delays Associated With New-Onset Seizures Revealing Dandy-Walker Variant

**DOI:** 10.7759/cureus.52802

**Published:** 2024-01-23

**Authors:** Sara Moudaffar, Mohssine Arraji, Bouchra Aabbassi, Iman Adali, Fatiha Manoudi

**Affiliations:** 1 Child and Adolescent Psychiatry, Ibn Nafis Hospital, University Hospital Center Mohammed VI, Marrakesh, MAR; 2 Child, Health and Development Research Laboratory, Faculty of Medicine and Pharmacy, Marrakesh, MAR; 3 Psychiatric Department, University Hospital Center Mohammed VI, Marrakesh, MAR

**Keywords:** new-onset seizure, dandy-walker variant, dandy-walker syndrome, posterior fossa malformations, developmental delay

## Abstract

Dandy-Walker malformation or syndrome is a rare congenital deformity in which the cerebellar vermis is hypoplastic and upwardly rotated, the fourth ventricle enlarged, and the posterior fossa cystically dilated. It represents the most common type of posterior fossa malformations that are usually diagnosed before the age of one year old. We present a seven-year-old boy with a history of neonatal hypotonia and delayed walking, who presented with speech and language difficulties. His physical examination and cognitive tests were unremarkable. The patient’s brain magnetic resonance imaging showed a partial defect of the inferior part of the cerebellar vermis and communication between a normal-sized cisterna magna and the fourth ventricle. There were no other coexisting central nervous system or systemic anomalies. This isolated inferior vermian hypoplasia was compatible with an uncommon variant of the Dandy-Walker syndrome. The aim of this report is to provide insight into the importance of implementing a pediatrician-psychiatrist collaboration in the clinical decision-making process of such developmental delay cases. What makes the present case further interesting are the new-onset unprovoked seizures that developed and recurred in the setting of such isolated and less severe posterior fossa anomaly, raising both diagnostic and therapeutic challenges.

## Introduction

Dandy-Walker malformation (DWM), also referred to as Dandy-Walker syndrome (DWS), is a rare complex malformation involving the posterior fossa and cerebellum, with an estimated incidence of one in 10,000 to 30,000 births [1,2]. DWM is defined by the classic triad of (1) complete or partial agenesis of the vermis; (2) enlargement of the posterior fossa with the upward displacement of the tentorium, transverse sinus, and torcular; and (3) cystic dilation of the fourth ventricle. How this entity can come about remains unclear, but it involves an intricate developmental anomaly of the cerebellar vermis [2,3]. Viral inflammatory disease contracted in utero, medication, alcohol, and diabetes have all been proposed as teratogenic agents. Most cases of DWM are sporadic with a poorly understood genetic background [1-3].

The diagnosis can be made antenatally, but the majority of cases present in infancy with early-onset macrocephaly (80% of cases), whereas older children may present with posterior fossa tumors [1-4]. Moderate to severe intellectual disability is common [4,5], and many cases have been associated with major psychopathology [1]. However, seizures are rarely reported, usually occurring in the setting of the classic DWM with coexisting central nervous system (CNS) malformations [1,6].

DWM represents the most common kind of a continuum spectrum of posterior fossa malformations. To our best knowledge, however, only four cases of the classic DWM have been documented in Morocco [7-10]. We provide a rare instance of a delayed diagnosis of a variant of DWM with significant speech and language delays and new-onset seizures in a seven-year-old Moroccan boy in this case report. The purpose of this report is also to highlight the importance of a thorough understanding of the patient’s medical history, a comprehensive psychiatric evaluation, and a multi-disciplinary approach to managing such complex developmental delay cases.

## Case presentation

This seven-year-old boy was referred by his general pediatrician to our outpatient child and adolescent psychiatric clinic due to an unexplained delayed speech and language (DSL) acquisition.

He was the second child of two brothers, born at term after an uneventful pregnancy to nonconsanguineous parents who denied any relevant personal medical history. His older brother was in healthy condition. Familial history was negative for neurodevelopmental disorders or epilepsy. His parents stated that he had experienced neonatal hypotonia, which had resolved on its own. He was not able to walk until the age of three and still had limited speech with only a few words. He had no history of headaches, vomiting, or other neurological issues. His physical examination was unremarkable; there were no dysmorphic features or abnormal neurological findings. A neuropsychological study revealed an average intelligence with no impairment of cognitive functions. Working with a speech-language therapist was strongly advised, with a plan to perform a brain magnetic resonance imaging (MRI) within two weeks.

A few days before the scheduled exam appointment, he was admitted to the emergency department suffering from multiple generalized tonic-clonic convulsions, followed by an onset of fever, breathlessness, and dry cough. Status epilepticus complicated by aspiration pneumonia was considered. Full blood count and serum electrolyte panel were normal, and routine electroencephalogram did not detect any interictal epileptiform activity. Besides high-flow oxygen, he was started on intravenous anticonvulsants and empirical antibiotics. Seizure control was achieved and pneumonia was improved.

**Figure 1:**
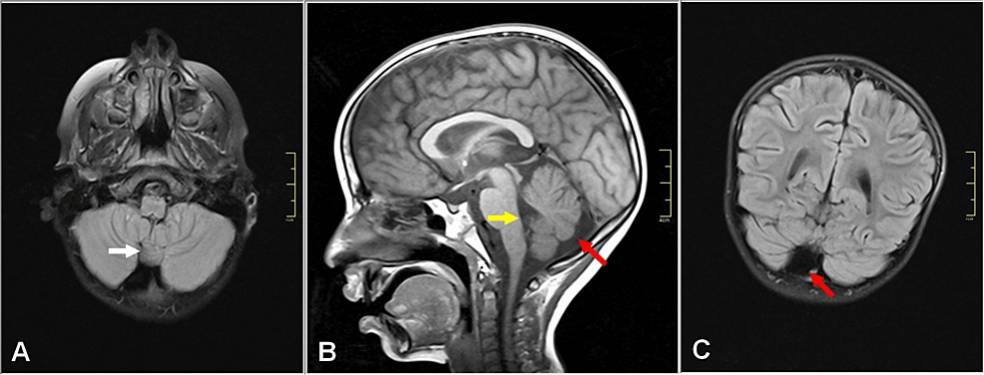
Brain MRI showing an inferior cerebellar vermian hypoplasia (white arrow), with communication between a normal-sized fourth ventricle (yellow arrow) and cisterna magna (red arrows). There was no hypoplasia, anterior rotation, upward displacement of the remaining vermis, or anterolateral displacement of the cerebellar hemispheres. The forebrain appeared normal. (A) Axial, (B) sagittal, and (C) coronal MRI images.

The brain MRI then revealed an isolated partial defect of the inferior part of the cerebellar vermis with communication between a normal-sized fourth ventricle and cisterna magna (Figure 1). The diagnosis of the Dandy-Walker variant (DWV) was considered.

The parents were told that this case is most likely sporadic and non-syndromic, which had not been confirmed through cytogenetic and molecular evaluation because of laboratory limitations. Although the child’s condition did not require surgical intervention, long-term anti-seizure monotherapy, and continued speech-language therapy were strongly recommended, whereupon significant progress in language acquisition had been made.

## Discussion

In this case, only a partial neuroimaging phenotype of DWM was noticed. Unlike individuals with the classic DWM, our patient had a small inferior defect of the vermis with neither torcular-lambdoid inversion nor posterior fossa expansion. These neuroimaging findings were congruent with diagnostic features of what is called DWV. To our knowledge, this is the first case of its kind to be reported in Morocco [7-10].

DWV is actually a term that was created to refer to a less severe constellation of DWS-like signs that do not meet the criteria for DWM [4]. Since then, however, it has led to some confusion as there has been no clinical separation clearly defined between these two entities. The term Dandy-Walker variant lacks specificity, creates confusion, and can lead to misdiagnosis and even incorrect genetic counseling [5,11,12]. Consequently, some authors would prefer to use a detailed anatomic description of our patient, and to consider this case as having an “isolated inferior vermian hypoplasia.”

Many patients affected by DWM can remain clinically asymptomatic for years, while individuals with DWV are more likely to present in adulthood than in infancy or childhood. However, the child in this case study presented with DSL that occurred in the setting of a past medical history significant for other motor disorders. Indeed, DWM/DWV has multiple neurodevelopmental complications since the cerebellum, which is the mainly affected structure, is the region of the brain that regulates movement coordination [1]. Had neonatal hypotonia and delayed walking not been ignored in this case, he would have been referred for a comprehensive diagnostic workup, including neuropsychological examinations and MRI, for early DSL identification and intervention. As the cerebellum could also modulate, although partially, cognition and behavior, our patient could have presented with concurrent intellectual disability and/or major psychopathology [1].

Seizures are rarely reported and are usually associated with the classic DWM plus additional supratentorial compartment anomalies [1,6]. In postnatal studies, the frequency of these associated CNS malformations ranges from 30% to 50% of cases of DWM/DWV, with ventriculomegaly and agenesis of the corpus callosum being the most common [13]. Of noteworthy is that new-onset unprovoked seizures are an unusual clinical presentation in the current patient with such isolated small inferior defect of the vermis.

There is currently a growing body of evidence to suggest the cerebellum is engaged during seizures and cerebellar impairments have been observed in patients with epilepsy [14]. Although pathophysiology is not well understood, cerebellar lesions, as in this case, may cause seizures due to loss of the inhibition of cortical areas or through intrinsic epileptic activity [15]. However, imaging evaluation of the forebrain is crucial to rule out other associated malformations, especially in the presence of atypical neurological signs, such as seizures.

The diagnosis of DWV and its associated CNS anomalies can be made based on anatomic findings that may be identified with postnatal ultrasonography or MRI as well as computed tomography, with MRI representing the “gold standard” [4,12]. In this case, however, it could have been difficult to solve prenatally the doubt of an isolated small inferior defect of the vermis. The antenatal sonography actually allows a definitive diagnosis of only the anatomic varieties on the more severe end of the disease spectrum in the Dandy-Walker continuum, those characterized by both a large posterior fossa cystic mass and a wide defect in the cerebellar vermis [16].

The present patient was considered on the lesser end of the disease spectrum in the Dandy-Walker continuum. Also known as the Dandy-Walker complex (DWC), this spectrum represents a continuum of posterior fossa anomalies that could range from mild (mega-cisterna magna only) to moderate (mild hypoplasia of vermis, enlarged fourth ventricle) to severe (agenesis of vermis, dilation of posterior fossa cyst and fourth ventricle) [4,17]. In this case, this wide disease spectrum expanded the differential diagnosis to also include mega-cisterna magna, posterior fossa arachnoid cyst, persistent Blake pouch cyst, fourth ventriculocele, and congenital vermian hypoplasia.

Degree of vermian and cerebellar hypoplasia, associated CNS and no CNS malformations, and coexisting genetic conditions have all been described as prognostic factors of DWM and its variants. More than 75% of patients with isolated inferior vermian hypoplasia have a favorable outcome. In some patients, mild functional deficits in fine motor activity and receptive language may be present [11,12], as in the current child. In addition, a favorable long-term outcome has been reported in 30% to 42% of infants with isolated posterior fossa abnormalities [18,19]. Besides seizures, the present case had proved to be of good prognosis, especially after maintaining a best-achieved walking acquisition at the age of three years with normal cognitive skills. However, genetic evaluation was strongly advised, as it may allow for better prognostication [19], especially to look at risks for a future pregnancy.

DWM/DWV may actually occur as part of a chromosomal abnormality or genetic syndrome in 50% of cases. In such a clinical scenario, recurrence risk may be high for first-degree relatives (i.e., siblings) [1,4]. As this case is considered to be sporadic non-syndromic, the recurrence risk is typically thought to be 1% to 10% for subsequent pregnancies [1,4,11]. Nonetheless, cytogenetic and molecular evaluation should have been performed, because aside from potential genetic factors, how this condition had arisen in this case is difficult to explain.

There are currently no formal guidelines on the surgical management of posterior fossa malformations. Asymptomatic patients should be followed without intervention. In a symptomatic patient, treatment changes according to the anatomical variant and associated anomalies. Hydrocephalus associated with DWS and the posterior fossa cyst can be surgically treated with cerebrospinal fluid (CSF) diversion [4]. It can be performed by neuroendoscopy, shunting procedures, or both [4,20]. Consequently, conservative management with anti-seizure medication and life-long physical and occupational therapy is the standard treatment option for the overall development and well-being of the current variant.

## Conclusions

DWV, or isolated inferior vermian hypoplasia, is a rare posterior fossa deformity. To our knowledge, no such report has been published in Morocco. In patients presenting with developmental delays, DWM/DWV must be considered in the differential diagnosis. For better prognostication, individuals diagnosed with such entity should be assessed for potential associated conditions, especially CNS anomalies in the presence of atypical neurological signs, such as seizures. However, this case favors the notion that seizures may develop without coexisting forebrain pathology even in a milder variant of the classic DWM. It also highlights the importance of implementing a pediatrician-psychiatrist collaboration in the clinical decision-making process of developmental delay cases (i.e., DSL). While surgery can be used in selected cases of DWC, conservative management is the standard treatment option for the variant found in our patient.
